# Bioinformatic identification of COLEC12 as a diagnostic biomarker and risk factor in pediatric FSGS

**DOI:** 10.3389/fped.2025.1539475

**Published:** 2025-04-01

**Authors:** Lei Gao, Zhenkun Yang, Yongbing Yang, Jingjing Wang, Wenyan Dong

**Affiliations:** ^1^ Department of Laboratory Medicine, Affiliated Children’s Hospital of Jiangnan University (Wuxi Children’s Hospital), Wuxi, China; ^2^ Department of Laboratory Medicine, The Affiliated Wuxi People’s Hospital of Nanjing Medical University, Wuxi, China; ^3^ Department of Anesthesiology, Affiliated Children’s Hospital of Jiangnan University (Wuxi Children’s Hospital), Wuxi, China

**Keywords:** pediatric FSGS, diagnosis biomarker, COLEC12, Nephroseq, GEO

## Abstract

**Background:**

In children, focal and segmental glomerulosclerosis (FSGS) and minimal change disease (MCD) have a similar appearance on electron microscopy, making them indistinguishable in clinical diagnosis. Here, we conducted genomic screening to identify a biomarker for the differential diagnosis and assess its clinical implications in pediatric FSGS.

**Methods:**

In the study, cohorts from public databases were analyzed through informatics analysis. First, molecular characteristics were analyzed using Gene Set Enrichment Analysis (GSEA) and Gene Ontology (GO) annotation, and Xcell was used to evaluate the infiltration of immune cells. Next, diagnostic and differentially diagnostic values of candidate biomarkers were studied using Receiver Operating Characteristic (ROC) curve analysis. Simultaneously, the clinical significance of the biomarker was analyzed in pediatric FSGS.

**Results:**

Compared to the healthy population, There were 27 genes significantly up-regulated and 129 genes significantly down-regulated in FSGS patients, which related to immune response and cell apoptosis. In FSGS, the T helper (Th) effector memory cell and macrophage M2 cell were significantly highly infiltrated. Meanwhile, regulatory T cells (Tregs) and plasma B cells showed a significant decrease. COLEC12 is the most different gene between FSGS and MCD, and had high value in the diagnosis and differential diagnosis of FSGS. In pediatric FSGS, COLEC12 expression is associated with poor prognosis.

**Conclusion:**

FSGS is intricately linked with immune responses. COLEC12 is a risk factor for FSGS in children and a candidate marker for FSGS diagnosis.

## Introduction

Focal segmental glomerulosclerosis (FSGS) is a major cause of pediatric nephrotic syndrome with an unclear mechanism, approximately 10% to 15% of pediatric FSGS cases progress to refractory kidney disease and end-stage renal disease (ESRD) (1). FSGS is a glomerular disease characterized by proteinuria and loss of glomerular filtration rate due to progressive sclerosis in glomeruli (2). Although clinical signs are suggestive, the confirmative diagnosis of FSGS is achieved only by histopathology findings. One of the key challenges in clinical practice is distinguishing primary FSGS from minimal change disease (MCD), as they share the same clinical presentations, including nephrotic-range proteinuria and hypoalbuminemia, as well as overlapping features of pathological manifestations (3). However, the later clinical course and renal prognosis of FSGS and MCD are often different. MCD frequently responds to corticosteroid therapy, and FSGS is more likely to exhibit steroid resistance and a higher risk of progression to chronic kidney disease (4). Therefore, finding a potential biomarker is crucial for early diagnosis, differentiation from MCD, and the development of effective therapeutic strategies.

In recent two decades, advances in molecular and genetic studies have revealed distinct biomarkers and pathogenic mechanisms underlying FSGS, which facilitate differential diagnosis and personalized treatment strategies. Apolipoprotein L1 (APOL1), the best-known gene, imparts a greatly increased risk of adult-onset FSGS and is associated with lower kidney function, more glomerulosclerosis and interstitial fibrosis, and greater propensity to progress to ESRD (5). Furthermore, soluble urokinase-type plasminogen activator receptor (suPAR), a circulating permeability factor, is described as inversely correlated with eGFR and having a deleterious effect on podocyte integrity (6). Several studies indicated that the concentration of suPAR was significantly higher in patients with primary FSGS than in those with MCD or membranous nephropathy (MN) and those in healthy control subjects (7, 8), suggesting suPAR as a potential diagnostic biomarker, but not substantiated by several studies (9, 10). The identification of clinically significant biomarkers is a complex process that faces multifaceted challenges encompassing ethical review requirements, sample size constraints, and financial limitations in longitudinal investigations. Public databases provide researchers a multi-center, large-scale platform to overcome these barriers, the high-throughput sequencing based on tissues (primary lesion, blood, urine, et al.) enabling systematic biomarker discovery through bioinformatics tools while mitigating resource-dependent biases. In this study, we aim to explore the molecular characteristics and potential diagnostic biomarkers for FSGS, providing insights into its differentiation from MCD. By leveraging genomic and bioinformatic approaches, we seek to improve diagnostic accuracy and inform targeted therapeutic interventions, particularly in pediatric populations where these diseases are prevalent yet diagnostically challenging.

## Materials and methods

### Data recourse and clinical information

In this study, the patient cohorts were obtained from Nephroseq (https://www.nephroseq.org). The related RNAseq datasets GSE200828 and GSE219185 were downloaded from Gene Expression Omnibus (GEO, http://www.ncbi.nlm.nih.gov/geo). Nephroseq is a platform that specifically integrates kidney disease genomics with clinical outcomes, enabling direct genotype-to-phenotype mining. GEO provides standardized, large-scale transcriptomic datasets across diverse populations, overcoming single-center sample limitations. These platforms are particularly vital for rare pediatric conditions like FSGS and MCD, where prospective cohort studies face ethical and caseload challenges. The dataset GSE200828 includes 153 kidney biopsy samples. In this study, we recruited 6 samples from healthy individuals, 31 samples from FSGS patients, and 19 samples from MCD patients for molecular characterization and differential gene expression analysis. The sequencing platform used was the Affymetrix Human Gene 2.1 ST Array and the results from each batch were normalized using the Robust Multichip Analysis (RMA) method. The dataset GSE219185 includes 220 kidney biopsy samples. In this study, we analyzed the correlation between COLEC12 expression and the clinical relevance of FSGS in a cohort of 28 pediatric patients. The study included pediatric patients aged 0-17 years with biopsy-confirmed primary FSGS and MCD, patients with secondary FSGS were excluded. Healthy individuals required normal renal function, absence of proteinuria, and no history of renal disease. The clinical information of the patients is shown in Table 1.

**Table 1:** Impact of COLEC12 expression on pediatric FSGS (*p < 0.05).

Patients characteristics	Low expression	High expression
Gender (Male/Female)	8/6	9/5
Age (years)	9.29 ± 4.84	12.29 ± 4.50
Proteinuria (Subnephrotic Proteinuria/Nephrotic Proteinuria)	9/5	5/8
Percent of Complete Remission*	85.7%	50.0%
Days from baseline to complete remission	655.50 ± 590.53	899.43 ± 638.22
Percent of ESRD or 40% EGFR loss	21.4% (3/11)	35.71% (5/11)
Days from baseline to ESRD or 40% EGFR Loss	1,195.78 ± 420.49	1,301.02 ± 454.55
GFR (CKD-EPI)	106. 70 ± 40.81	94.76 ± 34.43
Proteinuria at biopsy (mg/mg)	4.05 ± 5.38	5.93 ± 5.85

### Screening differentially expressed genes

We converted the probes into gene symbols in each dataset based on the platform's annotation file. For multiple probes mapped to the same gene symbol, the average value of those probes was used as the gene expression value. The analysis was performed in the R environment. Differentially expressed genes (DEGs) between the two groups were identified using the limma package, with the following criteria for adjustment: |Log_2_FoldChange|> 2 and adjust *p* value < 0.05. A volcano plot of the DEGs was generated using the ggplot2 package, while a heatmap was created using the pheatmap package. These biopsy-derived DEGs demonstrate translational potential as diagnostic biomarkers, serving as immunohistochemical markers, and could complement existing biomarkers in a multi-marker panel. Furthermore, through systematic validation of detectable abundances in blood and urine via subsequent large-scale cohort studies, these candidates may enable non-invasive monitoring through biochemical assays, thereby reducing the reliance on repeat tissue sampling in clinical practice.

### Gene set enrichment analysis (GSEA) and Go/KEGG functional annotation

GSEA was performed to identify pathways and biological processes significantly enriched in the dataset. Functional annotation of DEGs was conducted using Gene Ontology (GO) and Kyoto Encyclopedia of Genes and Genomes (KEGG) analyses to explore their biological roles and associated pathways. GSEA analyses were performed using the clusterProfiler package in R, with significance thresholds set at NES > 1 and NOM *p* value < 0.05. GO/KEGG functional annotation was performed on Metascape (https://metascape.org/). GO analysis included biological process (BP), cellular component (CC), and molecular function (MF) categories, while KEGG analysis focused on identifying key pathways associated with the DEGs.

### Estimation of the immune cellular composition of FSGS microenvironment

We used the patterns of expression of cell-type-specific genes to estimate the immune cellular composition. XCell is a gene signature-based method that integrates the advantages of gene set enrichment with deconvolution approaches, fitting RNA-seq and microarray data (11). In this study, cell-type enrichment analysis was performed using the XCell algorithm (https://xcell.ucsf.edu/).

### Statistical analysis

Statistical analyses were performed using SPSS version 23 and GraphPad Prism 8. The *t*-test was used to compare the means between two groups. The chi-square test was applied to compare clinical data between the two groups. Pearson correlation analysis was conducted to assess relationships between variables. A *p*-value of less than 0.05 was considered statistically significant for all analyses.

## Result

### Molecular and immune characteristics of FSGS

**Figure 1:**
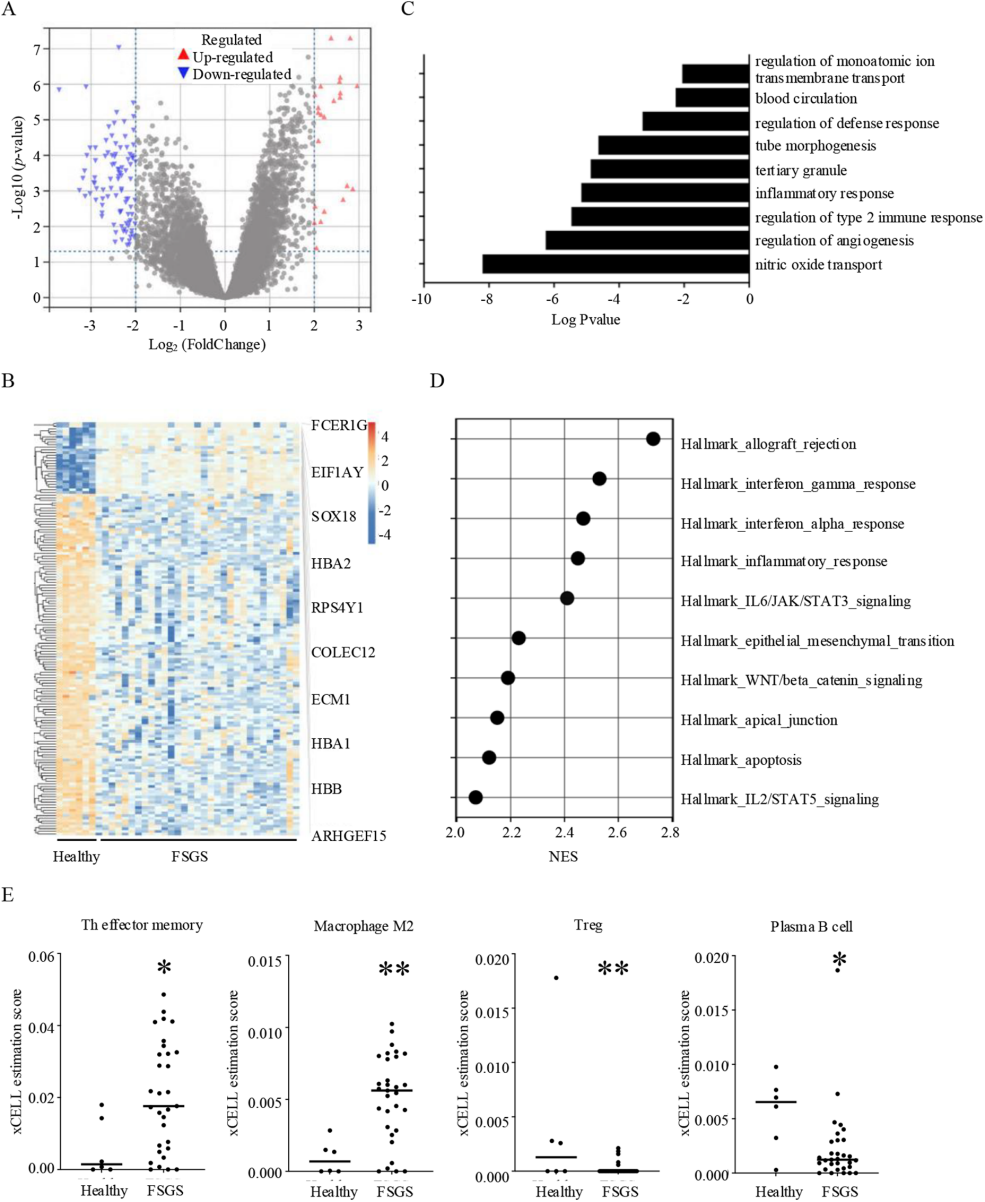
Molecular and immune characteristics of FSGS. **(A)** Volcano plot of differentially expressed genes in FSGS compare to healthy individuals with the criteria of |Log_2_FoldChange| > 2 and adjust *p* value < 0.05. **(B)** Heat map of differentially expressed genes between healthy individuals and FSGS, and top 10 up-regulated genes in FSGS. **(C)** GO and KEGG term analysis of up-regulated genes in FSGS. **(D)** GSEA analysis showing the top 10 most enriched genesets in FSGS with the criteria of NES > 2 and nom *p* value < 0.05. **(E)** Comparison of immune infiltration in renal microenvironment between healthy individuals and FSGS. *p < 0.05, **p < 0.01.

FSGS is a complex kidney disease characterized by distinct molecular and pathological features. Key molecular pathways implicated in FSGS include the activation of the renin-angiotensin-aldosterone system, aberrant TGF-β signaling, and increased oxidative stress, which contributes to podocyte injury and glomerular sclerosis (12, 13). In this study, we screened DEGs between healthy individuals and FSGS. Results showed that 27 genes were significantly up-expressed and 129 genes down-expressed in FSGS (Figure 1A). Among up expressed genes, FCER1G, EIF1AY, SOX18, HBA2, RPS4Y1, COLEC12, ECM1, HBA1, HBB, ARHGEF15 were the top10 genes with the most difference (Figure 1B; Supplementary Table S1). The significant GO/KEGG functional terms of the 27 up-expressed genes, including BP, MF, and CC, were illustrated in Figure 1C. The results indicated that nitric oxide transport, regulation of angiogenesis, regulation of type 2 immune response, and inflammatory response were significantly enriched by the GO-BP (Supplementary Table S2). The GSEA analysis showed that the allograft rejection pathway and inflammatory response (including IFN-α, IFN-γ, IL-2, and IL-6 signaling) were significantly enriched in FSGS. Additionally, results showed FSGS was also closely related to epithelial-mesenchymal transition (EMT), Wnt/β-catenin signaling, and apoptosis (Figure 1D). Immune cells are the mainstay of immune responses. Next, we analyzed the infiltration of immune cells in the renal microenvironment. Our results indicated that the T helper (Th) effector memory cell and macrophage M2 cell were significantly highly infiltrated. Meanwhile, regulatory T cells (Tregs) and plasma B cells showed a significant decrease in FSGS (Figure 1E). In summary, our results and published literature demonstrated that FSGS is a complex disease involving multiple mechanisms, particularly immune response and oxidative stress (13, 14).

### COLEC12 is a diagnostic and differential diagnostic biomarker for FSGS

**Figure 2:**
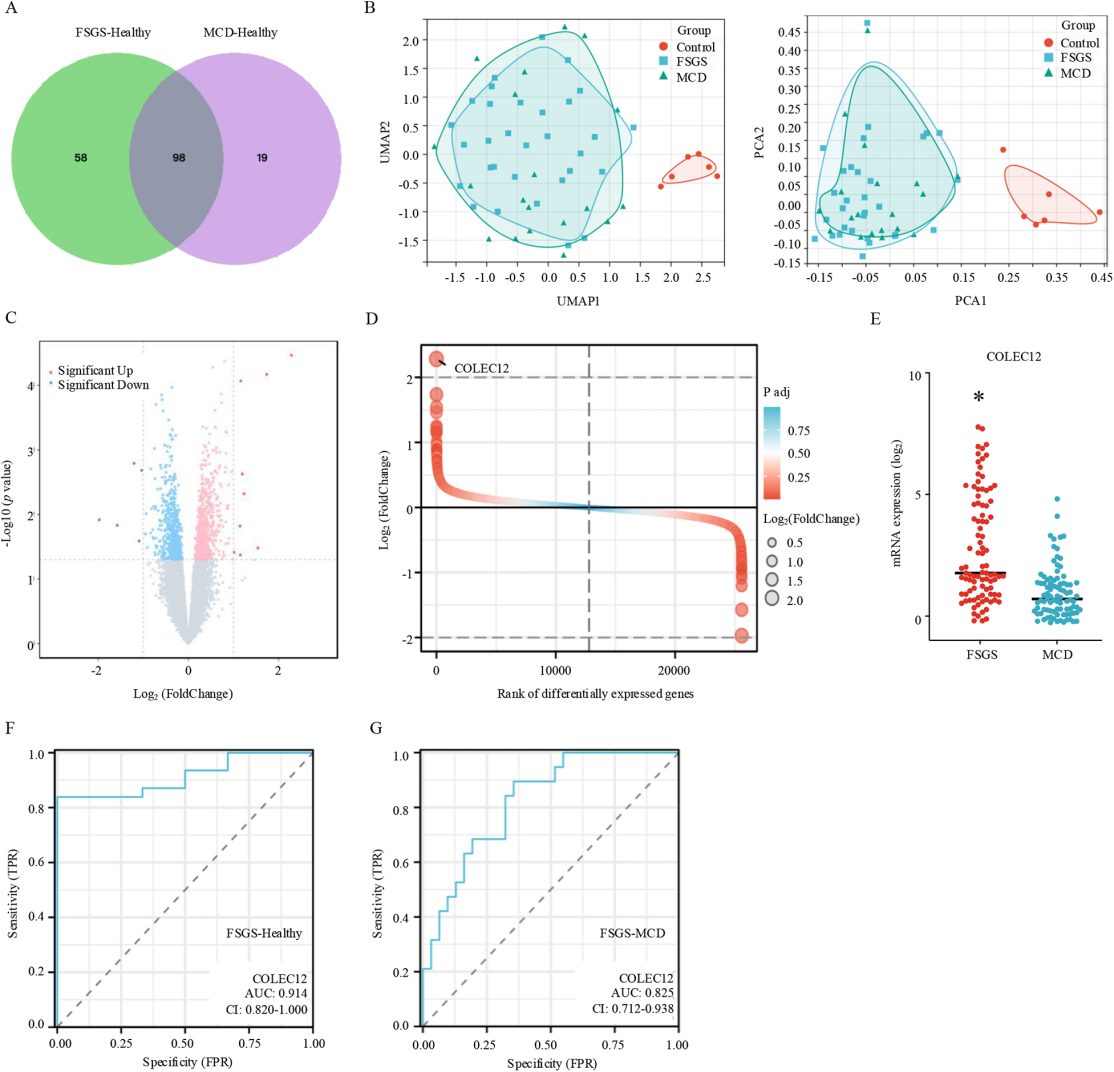
Diagnostic and differential diagnostic biomarker for FSGS. **(A)** Venn diagram of differentially expressed genes between MCD and FSGS. **(B)** UMAP (left) and PCA (right) plots for healthy individuals, MCD, and FSGS. **(C)** Volcano plot of differentially expressed genes in FSGS compare to MCD with the criteria of |Log_2_FoldChange| > 1 and adjust *p* value < 0.05. **(D)** Difference ranking chart of differentially expressed genes between MCD and FSGS. **(E)** The expression of COLEC12 between MCD and FSGS. *p < 0.05. **(F)** ROC plot reveals the diagnostic value of COLEC12 for FSGS. **(G)** ROC plot reveals the differential diagnostic value of COLEC12 for MCD and FSGS.

In clinical, the current diagnosis of FSGS and MCD, based on histopathologic features, have broadly overlapping clinical presentations and treatment approaches (15). First, we identified the DEGs between MCD and healthy individuals. The results revealed that 117 genes were differentially expressed between MCD and healthy individuals. Among these DEGs, 83.76% (98/117) were also differentially expressed in FSGS (Figure 2A). Furthermore, we conducted principal component analysis (PCA) and uniform manifold approximation and projection (UMAP) to analyze the intrinsic characteristics between healthy individuals, MCD, and FSGS. The results demonstrated that FSGS and MCD exhibit distinct characteristics compared to healthy individuals. However, FSGS and MCD share similar features, including overlapping molecular profiles (Figure 2B).

Next, we identified the DEGs between MCD and FSGS. Results showed that compared to MCD, 9 genes were significantly up-expressed and 5 genes down-expressed in FSGS (Figure 2C). Among the DEGs, COLEC12 was the most highly expressed gene in FSGS than MCD (Figures 2D,E). By using the receiver operating characteristic curve (ROC), the area under the ROC curve (AUC) was calculated by integrating the area under the ROC curve. AUC = 0.5 indicates no discrimination, 0.7 ≤ AUC < 0.8 indicates acceptable discrimination, 0.8 ≤ AUC < 0.9 indicates excellent discrimination, and AUC ≥ 0.9 indicates outstanding discrimination. In this study, the AUC for FSGS-healthy was 0.914, and the AUC for FSGS-MCD was 0.825, indicating COLEC12 was a potential biomarker for FSGS diagnosis (Figure 2F) and differential diagnosis of MCD and FSGS (Figure 2G).

### COLEC12 is associated with worse outcomes in pediatric FSGS

**Figure 3:**
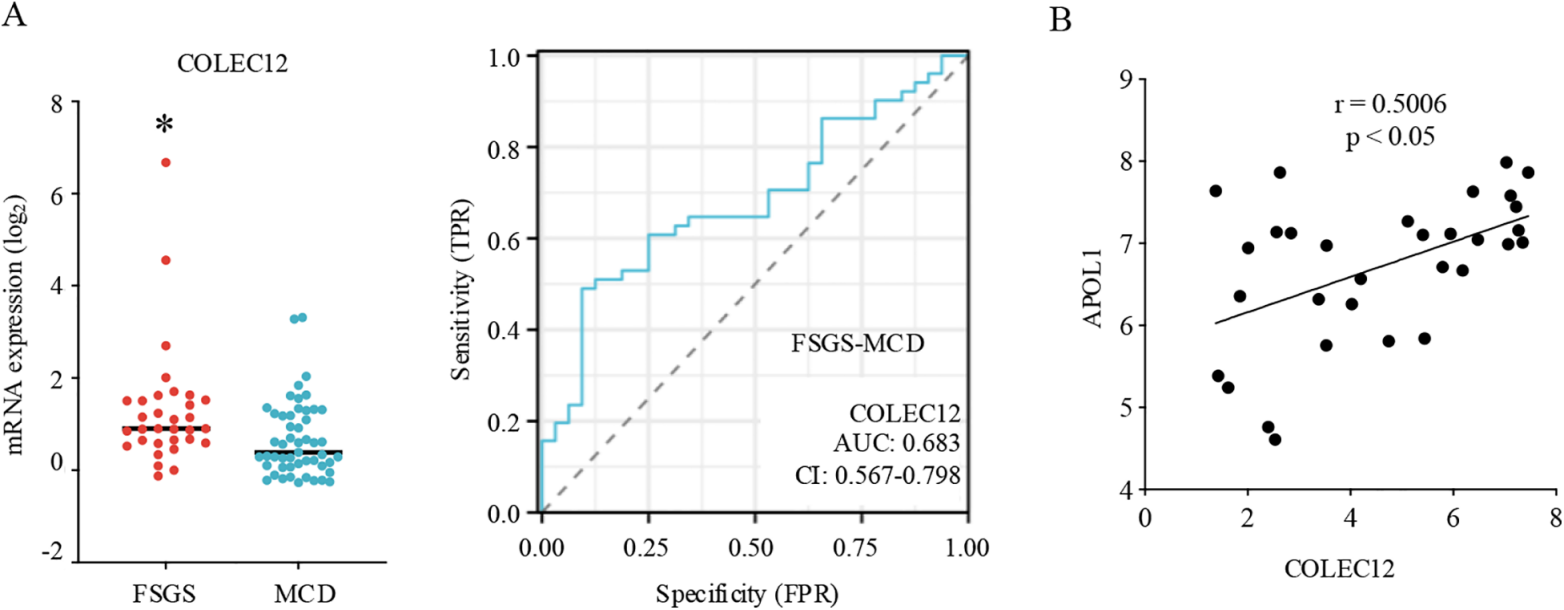
Clinical implications of COLEC12 in pediatric FSGS. **(A)** The expression and differential diagnostic value of COLEC12 between pediatric MCD and FSGS. *p < 0.05. **(B)** Correlation of COLEC12 and APOL1 in pediatric FSGS.

COLEC12 is a member of the C-lectin family that displays several functions related to host defense (16). In this study, we found that COLEC12 had a higher expression in FSGS than MCD and could differentiate FSGS from MCD in pediatric patients (Figure 3A). Additionally, the analysis of clinical information of pediatric patients revealed that FSGS occurs more often in male children than in female children, with a male-to-female ratio of approximately 1.54:1. However, neither gender nor age significantly correlated with COLEC12 expression. Further analysis revealed that COLEC12 expression is closely associated with the prognosis of FSGS. Children with low COLEC12 expression had a significantly higher rate of complete remission compared to those with high COLEC12 expression. Additionally, the time required to achieve complete remission was shorter in the low-expression group. Moreover, among children with high COLEC12 expression, 31.25% progressed to ESRD or 40% eGFR Loss, compared to 20% in the low-expression group (Table 1). APOL1 is a key pathological feature and is associated with an increased risk of FSGS. we investigated the correlation between COLEC12 and APOL1. Our study revealed a positive correlation between COLEC12 and APOL1 (Figure 3B), suggesting that COLEC12 plays an important role in the progression of pediatric FSGS.

## Discussion

FSGS is a leading glomerular cause of ESRD. Its clinical manifestations primarily include varying degrees of proteinuria and nephrotic syndrome. Pathologically, FSGS is characterized by focal and segmental glomerular sclerosis accompanied by podocyte foot process effacement (2). With the advancement of molecular biology and immunology, the understanding of FSGS has been further refined. In this study, we screened 27 genes that were significantly up-expressed and 129 genes down-expressed in FSGS compared to healthy individuals. Among these DEGs, FCER1G exhibited the most significant differential expression. FCER1G encodes the immunoglobulin Fcε receptor γ-subunit (FcRγ) and may modulate FSGS pathogenesis and progression through its role in mediating immune cell recognition of renal antigens and orchestrating subsequent immune activation cascades, particularly in podocyte injury patterns (17). Studies have shown that FSGS is closely associated with immune dysfunction. Our results indicated that the Th effector memory cells were significantly highly infiltrated in FSGS. The effector Th cells play crucial roles in various diseases. Research indicated that the level of effector Th cells-related cytokines such as IL-17 was highly expressed in renal tissues and induced podocyte apoptosis in an NF-κB-dependent manner (18). In this study, compared to healthy individuals, the complement system and the IL-6/JAK/STAT signaling pathway are activated in both the kidneys and peripheral blood cells of FSGS patients, and their activation levels are correlated with the severity of the disease (19, 20). IL-6 is an effector Th cells-related cytokine that plays multiple roles in immune regulation, inflammation, and other biological functions. In MCD and FSGS patients, multivariate analyses identified the levels of IL-6 as independent predictors of steroid resistance (21). Therefore, IL-6 blockade (e.g., siltuximab, tocilizumab) is a potential treatment approach for steroid-resistant FSGS. Li et al. demonstrated that tissue-resident memory T cells are also associated with the progression of FSGS. The cytokine IL-15 promotes the formation and activation of memory CD8^+^ *T* cells, which play a critical role in podocyte injury and glomerulosclerosis (22). In this study, through bioinformatics analysis, we found that inflammatory responses are closely associated with FSGS, with the IFN-α, IFN-γ, IL-2, and IL-6/JAK/STAT signaling pathways playing key roles.

FSGS is a pathological alteration, and its diagnosis primarily relies on tissue biopsy. MCD and FSGS are common pathological types of nephrotic syndrome in children and adults. Both of them exhibit significant podocyte foot process effacement. In the early stages, FSGS and MCD are often difficult to differentiate morphologically (23). In this study, we also indicated that FSGS and MCD share similar molecular characteristics and cannot be differentiated through UMAP and PCA dimensionality reduction clustering. Therefore, identifying diagnostic biomarkers for FSGS, especially molecular markers for differential diagnosis, is of paramount importance. CD80 is an essential co-stimulatory factor for T lymphocyte activation. In FSGS patients, CD80 expression on podocytes is significantly upregulated, making it a potential molecular marker for the diagnosis of FSGS (24). However, studies have shown that CD80 is elevated to varying degrees in patients with active kidney disease. Therefore, its role as a differential diagnostic marker requires further research and validation (25). Other studies have shown that the differentiation antigen CD44 is highly expressed in the glomerular parietal epithelial cells in FSGS patients, whereas it is not expressed in MCD patients. This suggests that CD44 could serve as a reliable pathological feature for distinguishing between MCD and FSGS (26). In this study, RNA sequencing results from kidney biopsy tissue were used to identify differential genes between FSGS and healthy individuals, and a comparison was performed with the differential genes in MCD. This approach led to the identification of molecular markers for FSGS diagnosis and differential diagnosis. The results revealed that COLEC12 is the most significantly differentially expressed molecule between FSGS and MCD, demonstrating high value in both the diagnosis and differential diagnosis of FSGS. In clinical practice, COLEC12 could serve as an immunohistochemical marker to complement existing diagnostic markers in a multi-marker panel. Furthermore, through systematic validation of detectable abundances in blood and urine via subsequent large-scale cohort studies, COLEC12 may enable non-invasive monitoring through biochemical assays, thereby reducing the reliance on repeat tissue sampling.

COLEC12 is a member of the C-type lectin family and is closely involved in regulating immune response (16, 27). Research indicated that COLEC12 was established as an effective rheumatoid arthritis diagnostic marker, and was highly positively correlated with T cells follicular helper infiltration and highly negatively correlated with the expression of mast cells (28). However, the role of COLEC12 in pediatric FSGS has not been reported previously. The results of this study show that COLEC12 expression is closely associated with the prognosis of FSGS. Additionally, COLEC12 expression is positively correlated with the expression of the FSGS risk factor APOL1. APOL1 coding variants, termed G1 and G2, are established genetic risk factors for kidney disease, in individuals of African ancestry (29). However, only some individuals with APOL1 high-risk genotypes have CKD or kidney failure, indicating genetic background contributes to the APOL1 high-risk kidney disease relationship. In this study, demographic metadata, particularly ethnicity, were unavailable, raising concerns about generalizability given the COLEC12-APOL1 correlation and APOL1's established association with African ancestry populations.

This study has several limitations. The bioinformatics analysis relied on public datasets from Nephroseq and GEO with relatively small sample sizes, particularly in pediatric cohorts, which may limit statistical power and generalizability. In addition, while *post-hoc* power analysis indicated adequate statistical power (*α* = 0.05, effect size = 0.9; ∼80%), prospective validation in larger, multi-ethnic cohorts is essential to confirm diagnostic robustness. Future work should prioritize translational validation, including assessing COLEC12 expression in blood/urine for non-invasive diagnostics and exploring its utility in diverse populations. Lastly, integrating COLEC12 into existing diagnostic algorithms and investigating targeted therapies could enhance clinical applicability. Addressing these limitations would strengthen the biomarker's diagnostic validity and therapeutic relevance in pediatric FSGS.

## Data Availability

Publicly available datasets were analyzed in this study. This data can be found here: https://www.ncbi.nlm.nih.gov/geo/ GSE200828, GSE219185.
